# Supplementary material for: Comparison of Volatiles in Different Jasmine Tea Grade Samples Using Electronic Nose and Automatic Thermal Desorption-Gas Chromatography-Mass Spectrometry Followed by Multivariate Statistical Analysis
Source: Molecules. 2020 Jan 16;25(2):380. doi: 10.3390/molecules25020380 (PMC7024305; doi:10.3390/molecules25020380)
Supplement: Supplementary file 1 [file molecules-25-00380-s001.zip › Supplementary files/Fig. S2.docx]

|   **1G** |  |
| --- | --- |
|   **2G** |   **5G**  **4G** |
|   **3G** |   **6G** |

**Fig. S2.** Total ion chromatogram diagram of VOCs with different grades jasmine tea
