# Supplementary material for: Comparison of Volatiles in Different Jasmine Tea Grade Samples Using Electronic Nose and Automatic Thermal Desorption-Gas Chromatography-Mass Spectrometry Followed by Multivariate Statistical Analysis
Source: Molecules. 2020 Jan 16;25(2):380. doi: 10.3390/molecules25020380 (PMC7024305; doi:10.3390/molecules25020380)
Supplement: Supplementary file 1 [file molecules-25-00380-s001.zip › Supplementary files/Tab. S1.docx]

**Tab. S1**. Description of aroma characteristic in each standard jasmine tea sample

| Standard jasmine tea grade sample | Description of sensory feature in aroma |
| --- | --- |
| 1G | Extremely fresh, strong and lasting |
| 2G | Extremely fresh and strong |
| 3G | Fresh and strong |
| 4G | Strong |
| 5G | Slight lack of aroma intensity |
| 6G | Insufficient in aroma intensity |
