# Supplementary material for: Comparison of Volatiles in Different Jasmine Tea Grade Samples Using Electronic Nose and Automatic Thermal Desorption-Gas Chromatography-Mass Spectrometry Followed by Multivariate Statistical Analysis
Source: Molecules. 2020 Jan 16;25(2):380. doi: 10.3390/molecules25020380 (PMC7024305; doi:10.3390/molecules25020380)
Supplement: Supplementary file 1 [file molecules-25-00380-s001.zip › Supplementary files/Tab. S2.docx]

**Tab. S2.** Gas sensors array and corresponding volatile components

| **Gas sensor** | **Volatile components of response** |
| --- | --- |
| S1 | Ammonia and Amines |
| S2 | Hydrogen sulfide and sulfide |
| S3 | Hydrogen |
| S4 | Alcohol and Organic Solvent |
| S5 | Volatile gases in food cooking |
| S6 | Methane, ethane and hydrocarbons |
| S7 | Flammable gases |
| S8 | Volatile Organic Compounds |
| S9 | Hydroxide, gasoline and kerosene |
| S10 | Alkanes and flammable gases |
