# Supplementary material for: Comparison of Volatiles in Different Jasmine Tea Grade Samples Using Electronic Nose and Automatic Thermal Desorption-Gas Chromatography-Mass Spectrometry Followed by Multivariate Statistical Analysis
Source: Molecules. 2020 Jan 16;25(2):380. doi: 10.3390/molecules25020380 (PMC7024305; doi:10.3390/molecules25020380)
Supplement: Supplementary file 1 [file molecules-25-00380-s001.zip › Supplementary files/Tab. S3.docx]

| **Tab. S3.** VIP score of identified VOCs | | |
| --- | --- | --- |
| No. | **Var ID (Primary)** | **VIP Value** |
| 1 | Phenylethyl Alcohol | 1.61567 |
| 2 | Benzoic acid ethyl ester | 1.44024 |
| 3 | (Z)-2-Hexenyl acetate | 1.39582 |
| 4 | (Z)-3-Hexenyl angelate | 1.34945 |
| 5 | alpha.-Terpinene | 1.34762 |
| 6 | (Z)-beta.-Ocimene | 1.2818 |
| 7 | beta.-Cadinene | 1.2713 |
| 8 | beta.-Cubebene | 1.2546 |
| 9 | gamma.-Muurolene | 1.24311 |
| 10 | (Z)-3-Hexenyl benzoate | 1.19167 |
| 11 | Germacrene D | 1.186 |
| 12 | (Z)-3-Hexenyl 2-methylbutanoate | 1.14285 |
| 13 | alpha.-Muurolene | 1.13621 |
| 14 | benzyl propionate | 1.1236 |
| 15 | Myrcene | 1.11678 |
| 16 | 2-Phenethyl acetate | 1.11479 |
| 17 | g-Cadinene | 1.10775 |
| 18 | (Z)-3-Hexenyl acetate | 1.08812 |
| 19 | Nerolidol | 1.07855 |
| 20 | Butyl benzoate | 1.07428 |
| 21 | alpha.-Patchoulene | 1.07373 |
| 22 | Indole | 1.06786 |
| 23 | gamma.-Cadinene | 1.06748 |
| 24 | Geraniol | 1.06402 |
| 25 | 3-Hexen-1-ol | 1.04551 |
| 26 | alpha.-paleolene | 1.03871 |
| 27 | Methyl anthranilate | 1.03383 |
| 28 | alpha.-Caryophyllene | 1.0168 |
| 29 | alpha.-Elemene | 1.01178 |
| 30 | alpha.-Terpineol | 1.0042 |
| 31 | ethyl salicylate | 0.984532 |
| 32 | 5-Hepten-2-one | 0.950174 |
| 33 | Naphthalene | 0.945987 |
| 34 | Acetophenone | 0.944677 |
| 35 | Methyl benzoate | 0.929694 |
| 36 | alpha.-Farnesene | 0.922482 |
| 37 | (Z)-3-Hexenyl butyrate | 0.921303 |
| 38 | beta.-Elemene | 0.90717 |
| 39 | alpha.-Cadinol | 0.881591 |
| 40 | Eugenol | 0.868278 |
| 41 | Benzyl butyrate | 0.852741 |
| 42 | Caryophyllene | 0.842562 |
| **Tab. S2** (continued) | |  |
| No. | **Var ID (Primary)** | **VIP Value** |
| 43 | (-)-Terpinen-4-ol | 0.840178 |
| 44 | 2-Methylnaphthalene | 0.825714 |
| 45 | Cyclopentanol | 0.818385 |
| 46 | Benzyl Benzoate | 0.806168 |
| 47 | (E,E)-2,4-Heptadienal | 0.80144 |
| 48 | alpha.-Copaene | 0.799922 |
| 49 | (Z)-3-Hexenyl (Z)-3-hexenoate | 0.797151 |
| 50 | Decanal | 0.784413 |
| 51 | Benzyl acetate | 0.778721 |
| 52 | Acetic acid, 2-ethylhexyl ester | 0.763542 |
| 53 | beta.-Cyclocitral | 0.755648 |
| 54 | 1-Hexanol | 0.755621 |
| 55 | Benzaldehyde | 0.746558 |
| 56 | Linalool | 0.738395 |
| 57 | alpha.-Cubebene | 0.738152 |
| 58 | Hexanal | 0.718741 |
| 59 | Benzyl alcohol | 0.717701 |
| 60 | Methyl salicylate | 0.712298 |
| 61 | alpha.-Pinene | 0.682756 |
| 62 | Linalool oxide | 0.680292 |
| 63 | (Z)-Linalool oxide | 0.66671 |
| 64 | Limonene | 0.654732 |
