# Supplementary material for: Comparison of Volatiles in Different Jasmine Tea Grade Samples Using Electronic Nose and Automatic Thermal Desorption-Gas Chromatography-Mass Spectrometry Followed by Multivariate Statistical Analysis
Source: Molecules. 2020 Jan 16;25(2):380. doi: 10.3390/molecules25020380 (PMC7024305; doi:10.3390/molecules25020380)
Supplement: Supplementary file 1 [file molecules-25-00380-s001.zip › Supplementary files/Tab. S4.docx]

| **Tab. S4. VOCs of grade jasmine tea** | | | | | | |
| --- | --- | --- | --- | --- | --- | --- |
| **No.** | **Compound** | **Correlation^g^** | **Source** | **Reference** | **Odor note** | **Reference** |
|  |  |  |  |  |  |  |
|  | **Alcohols** |  |  |  |  |  |
| 1 | Cyclopentanol | Negative | LongJing tea | Guo et al. (2016) | / | / |
| 2 | 1-Hexanol | Negative | / | / | Fruity | Xu et al. (2016) |
| 3 | Benzyl alcohol | / | Flower, base tea | Chen et al. (2017), Edris et al. (2008) | Sweet, roasted, mild | Li et al. (2018), Wang et al. (2017) |
| 4 | (Z)-Linalool oxide | Negative | (Sen-cha) | Jumtee et al. (2011) | Floral, wood, green, | Zhou et al. (2019), Wang et al. (2017) |
| 5 | (E)-Linalool oxide | Negative | (Sen-cha) | Jumtee et al. (2012) | Floral, wood, fruity | Zhou et al. (2019), Wang et al. (2017) |
| 6 | 3-Hexen-1-ol | / | Flower | Chen et al. (2017), Edris et al. (2008) | Green, lettuce-like | Alasalvar et al. (2012), Zhou et al. (2019) |
| 7 | Linalool | / | Flower, base tea | Chen et al. (2017), Yu et al.(2017) | Floral, fruity, wood | Wang et al. (2017), Zhou et al. (2019) |
| 8 | Phenylethyl Alcohol | / | Flower | Chen et al. (2017) | Sweet, rose-like | Li et al. (2018), Qin et al. (2013) |
| 9 | (-)-Terpinen-4-ol | / | / | / | / | / |
| 10 | alpha.-Terpineol | / | Flower | Chen et al. (2017) | mint, sweet, floral | Wu et al. (2013), Xu et al. (2016) |
| 11 | Geraniol | / | Flower | Chen et al. (2017) | Rose, geranium | Li et al. (2018), Zhou et al. (2019) |
| 12 | Nerolidol | / | / | / | Floral, Green | Li et al. (2018), Zhou et al. (2019) |
| 13 | alpha.-Cadinol | / | Flower | Chen et al. (2017) | / | / |
|  | **Aldehydes** |  |  |  |  |  |
| 14 | Hexanal | Negative | Matcha | Baba et al. (2017) | Green, fruity, tallow | Li et al. (2018), Wang et al. (2017) |
| 15 | Benzaldehyde | Negative | base tea | Chen et al. (2017), Edris et al. (2008) | Almond, sugar, burnt | Li et al. (2018), Zhou et al. (2019) |
| 16 | (E,E)-2,4-Heptadienal | Negative | Matcha | Baba et al. (2017) | Nut, fat, floral | Li et al. (2018), Wang et al. (2017) |
| 17 | Decanal | Negative | Xinyang Maojian | Long et al. (2019) | Herbal, fatty, citrus | Li et al. (2018), Zhou et al. (2019) |
| 18 | beta.-Cyclocitral | Negative | Xinyang Maojian | Long et al. (2019) | Mint, fruit, green,mild | Li et al. (2018), Wang et al. (2017) |
|  | **Esters** |  |  |  |  |  |
| 19 | (Z)-3-Hexenyl acetate | Positive | Flower | Chen et al. (2017), Yu et al.(2017) | Green,banana-like | Zhou et al. (2019) |
| 20 | (Z)-2-Hexenyl acetate | / | / | / | / | / |
| 21 | Methyl benzoate | Positive | / | Edris et al. (2008) | Floral | / |
| 22 | Acetic acid, 2-ethylhexyl ester | / | / | / | / | / |
| 23 | Benzyl acetate | Positive | Flower | Chen et al. (2017), Yu et al.(2017) | Floral, fruity | Zhou et al. (2019) |
| 24 | Benzoic acid ethyl ester | Positive | Flower | Chen et al. (2017) | / | / |
| 25 | (Z)-3-Hexenyl butyrate | Positive | Flower | Chen et al. (2017) | Fruity | Li et al. (2018) |
| 26 | Methyl salicylate | Positive | Flower | Chen et al. (2017), Yu et al.(2017) | Sweet, spicy, minty | Li et al. (2018), Zhou et al. (2019) |
| 27 | (Z)-3-Hexenyl 2-methylbutanoate | Positive | Flower | Chen et al. (2017) | / | / |
| 28 | 2-Phenethyl acetate | / | / | Edris et al. (2008) | Peach | Li et al. (2018) |
| 29 | Benzyl propionate | / | / | / | / | / |
| 30 | Ethyl salicylate | Positive | Flower | Chen et al. (2017) | / | / |
| 31 | (Z)-3-Hexenyl angelate | / | / | / | / | / |
| 32 | Methyl anthranilate | / | Flower | Chen et al. (2017), Edris et al. (2008) | Peach, sweet, fruity | Li et al. (2018), Zhou et al. (2019) |
| 33 | Benzyl butyrate | Positive | / | / | Apricot, jasmine | Li et al. (2018) |
| 34 | Butyl benzoate | Positive | / | Edris et al. (2008) | Sweet, floral | Li et al. (2018) |
| 35 | (Z)-3-Hexenyl (Z)-3-hexenoate | Positive | / | / | / | / |
| 36 | (Z)-3-Hexenyl benzoate | Positive | Flower | Chen et al. (2017), Yu et al.(2017) | Green, spicy, wood | Li et al. (2018), Wang et al. (2017) |
| 37 | Benzyl Benzoate | / | Flower | Chen et al. (2017) | Sweet, balsamic | Li et al. (2018) |
|  | **Hydrocarbons** |  |  |  |  |  |
| 38 | alpha.-Pinene | Negative | / | / | / | / |
| 39 | Myrcene | Positive | Flower | Chen et al. (2017) | Orange, sweet, floral | Li et al. (2018), Xu et al. (2016) |
| 40 | alpha.-Terpinene | Positive | / | / | Woody | Xu et al. (2016) |
| 41 | Limonene | Negative | / | / | Fruity | Xu et al. (2016) |
| 42 | (Z)-beta.-Ocimene | Positive | Flower | Chen et al. (2017), Yu et al.(2017) | Herbaceous | Wang et al. (2017) |
| 43 | alpha.-Elemene | Positive | / | Yu et al.(2017) | / | / |
| 44 | alpha.-Cubebene | / | Flower | Chen et al. (2017) | / | / |
| 45 | alpha.-Copaene | / | Flower, base tea | Chen et al. (2017) | / | / |
| 46 | Germacrene D | Positive | Flower | Chen et al. (2017), Edris et al. (2008) | / | / |
| 47 | gamma.-Cadinene | / | / | / | / | / |
| 48 | beta.-Elemene | / | Flower | Chen et al. (2017), Edris et al. (2008) | / | / |
| 49 | alpha.-paleolene | / | / | / | / | / |
| 50 | Caryophyllene | / | Flower, base tea | Chen et al. (2017) | Floral, wood | Li et al. (2018), Wang et al. (2017) |
| 51 | beta.-Cubebene | Positive | / | / | / | / |
| 52 | alpha.-Caryophyllene | / | Flower, base tea | Chen et al. (2017) | / | / |
| 53 | gamma.-Muurolene | Positive | Flower | Chen et al. (2017), Yu et al.(2017) | / | / |
| 54 | alpha.-Farnesene | Positive | Flower | Chen et al. (2017), Yu et al.(2017) | Floral, herbaceous | Li et al. (2018), Wang et al. (2017) |
| 55 | (-)-g-Cadinene | Positive | Flower | Chen et al. (2017) | / | / |
| 56 | beta.-Cadinene | Positive | Flower | Chen et al. (2017) | / | / |
| 57 | alpha.-Muurolene | Positive | Flower | Chen et al. (2017) | Fruity | Li et al. (2018) |
| 58 | alpha.-Patchoulene | / | / | / | / | / |
| 59 | Naphthalene | / | base tea | Chen et al. (2017) | / | / |
| 60 | 2-Methylnaphthalene | / | / | / | / | / |
|  | **Ketones** |  |  |  |  |  |
| 61 | 6-Methyl-5-hepten-2-one | Negative | / | / | Sweet, fruity, orange | Qin et al. (2013), Xu et al. (2016) |
| 62 | Acetophenone | / | / | / | / | / |
|  | **Nitrogenous compounds** |  |  |  |  |  |
| 63 | Indole | Positive | Flower | Chen et al. (2017), Edris et al. (2008) | Nutty, floral, burnt | Li et al. (2018), Zhou et al. (2019) |
|  | **Phenols** |  |  |  |  |  |
| 64 | Eugenol | / | / | / | Clove, spicy | Wang et al. (2017) |
|  |  |  |  |  |  |  |
|  |  |  |  |  |  |  |
| ^g^Correlation between content and quality grade in literature reports | | | | | |  |
