# Supplementary material for: Comparison of Volatiles in Different Jasmine Tea Grade Samples Using Electronic Nose and Automatic Thermal Desorption-Gas Chromatography-Mass Spectrometry Followed by Multivariate Statistical Analysis
Source: Molecules. 2020 Jan 16;25(2):380. doi: 10.3390/molecules25020380 (PMC7024305; doi:10.3390/molecules25020380)
Supplement: Supplementary file 1 [file molecules-25-00380-s001.zip › Supplementary files/Fig. S1.doc]

**Fig. S1.** The processing steps of standard Chinese jasmine tea grade samples
